# Association of PPP1R13L and CD3EAP polymorphisms with risk and survival of non-small cell lung cancer in Chinese non-smoking females

**DOI:** 10.18632/oncotarget.20224

**Published:** 2017-08-12

**Authors:** Xu Feng, Xue Fang, Lingzi Xia, Yangwu Ren, Xuelian Li, Xiaowei Quan, Baosen Zhou

**Affiliations:** ^1^ Department of Epidemiology, School of Public Health, China Medical University, Liaoning, China; ^2^ Department of Health Statistics, School of Public Health, Shenyang Medical College, Liaoning, China; ^3^ Key Laboratory of Cancer Etiology and Prevention (China Medical University), Liaoning Provincial Department of Education, Liaoning, China

**Keywords:** PPP1R13L, CD3EAP, single nucleotide polymorphism, non-small cell lung cancer

## Abstract

PPP1R13L and CD3EAP were confirmed to play important roles in transcription and apoptosis. SNPs in PPP1R13L and CD3EAP may be associated with lung cancer risk and survival. This study investigated the association of PPP1R13L rs1005165 and CD3EAP rs967591 with non-small cell lung cancer (NSCLC) risk and survival in Chinese non-smoking females. 442 NSCLC cases and 480 cancer-free controls were conducted in the case-control study, and 283 cases were in cohort study. Genotype was determined by Taqman real-time PCR. The statistical analyses were performed by SPSS 22.0 software. We found that rs1005165 and rs967591 were significantly associated with NSCLC risk in Chinese non-smoking females. For rs1005165, compared with homozygous wild CC genotype, carriers of CT or TT genotype had lower risk of NSCLC (adjusted ORs were 0.675 and 0.713, 95% CI were 0.461-0.988 and 0.525-0.968, respectively), adjusted OR for dominant model was 0.702, 95% CI was 0.526-0.937. For rs967591, AA genotype (adjusted OR = 0.721, 95% CI = 0.532-0.978) and at least one A allele (GA+AA) (adjusted OR = 0.716, 95% CI = 0.536-0.956) were significantly correlated with lower risk of NSCLC, compared with GG genotype. But we didn’t find correlation between the two SNPs and survival time in Chinese non-smoking NSCLC females. In general, we found PPP1R13L rs1005165 and CD3EAP rs967591 might be associated with lower NSCLC risk in Chinese non-smoking females, but no significant relationship was found with NSCLC survival.

## INTRODUCTION

Lung cancer is the leading cause of cancer-related risk and mortality worldwide. There were 1.8 million new diagnosed lung cancer patients in 2012 and 1.59 million patients were dead because of lung cancer [1]. Epidemiological studies have confirmed that smoking was one of the risk factors for lung cancer, but only 20% of smokers will develop lung cancer [2]. About 53% female lung cancer patients are non-smokers [3], which suggest that genetic and environmental factors may have effect on lung cancer risk and development. About 80% of lung cancers are non-small cell lung carcinomas (NSCLC), and the 5-year survival rate of NSCLC is less than 20% [4]. Therefore, it is meaningful to explore the factors that may be associated with NSCLC risk or survival in non-smoking females.

Protein phosphatase 1, regulatory subunit 13 like (PPP1R13L) and CD3e molecule, epsilon associated protein (CD3EAP) are both in a small region of human chromosome 19q13.3. PPP1R13L protein is an oncogene [5-7]. It can bind wide-type p53 to inhibit apoptosis [8] and bind to the NF-kappaB subunit p65 (RelA) to inhibit its transcriptional activity [9]. Overexpression of PPP1R13L protein can inhibit p53-mediated apoptosis in response to treatment with cisplatin [10] and was confirmed to be an unfavorable prognostic factor in NSCLC patients [11]. CD3EAP is anti-sense to ERCC1 and overlaps with ERCC1. It is suggested to bind the transcript of ERCC1 to inhibit translation and indicate a function on ribosomal RNA transcription [12]. A study suggested that the gene product of CD3EAP might transduce activation signal for interleukin-2 production in T cells [13]. The expression of CD3EAP may be related to cell proliferation [14]. These studies revealed that PPP1R13L and CD3EAP played important roles in carcinogenesis and prognosis. Several single nucleotide polymorphisms (SNPs) in the two genes have already been identified to be associated with the risk or survival of cancer [15-17]. Nonetheless, relatively few studies have investigated the relationship of SNPs in PPP1R13L and CD3EAP and risk or survival of NSCLC. In this study, whether PPP1R13L rs1005165 and CD3EAP rs967591 may associate with NSCLC risk and survival in Chinese non-smoking females was evaluated.

## RESULTS

### Characteristics of study population in case-control study

A total of 442 NSCLC patients and 480 cancer-free controls were included in the case-control study, who were all non-smoking females. The mean ages were 56.42±11.71 and 56.94±13.34 in case group and control group, respectively. There was no significant difference between two groups (t = 0.625, *P* = 0.532). Among the cases, there were 349 adenocarcinomas patients and 93 squamous cell carcinomas patients.

### Polymorphisms and NSCLC risk

Genotyping was successful for 905 subjects of rs1005165 and 906 subjects of rs967591. All allele distributions in control groups were consistent with Hardy-Weinberg equilibrium (*P*=0.19 for rs1005165, and *P*=0.06 for rs967591).

Genotype distribution and association with NSCLC risk were shown in Table 1. For rs1005165, CT and TT genotype carriers had lower risk of NSCLC compared with homozygous wild genotype CC genotype carriers (adjusted OR = 0.675, 95% CI = 0.461-0.988 and adjusted OR = 0.713, 95% CI = 0.525-0.968, respectively). The adjusted OR for rs1005165 dominant model was 0.702, 95% CI was 0.526-0.937. Taking rs967591 GG as reference, GA genotype and GA+AA dominant model showed a lower risk of NSCLC, and adjusted OR were 0.721 (0.532-0.978) and 0.716 (0.536-0.956), respectively.

**Table 1:** Distribution of genotypes and ORs for NSCLC cases and controls

SNP	Cases (%)	Controls (%)	OR (95% CI)^a^	*P*
rs1005165				
CC	145 (33.3)	119 (25.3)	Ref.	-
CT	209 (48.0)	249 (53.0)	0.675 (0.461-0.988)	0.043^*^
TT	81 (18.6)	102 (21.7)	0.713 (0.525-0.968)	0.030^*^
CT+TT	290 (66.7)	351 (74.7)	0.702 (0.526-0.937)	0.016^*^
rs967591				
GG	142 (32.9)	120 (25.3)	Ref.	-
GA	212 (49.1)	257 (54.2)	0.721 (0.532-0.978)	0.035^*^
AA	78 (18.1)	97 (20.5)	0.704 (0.478-1.036)	0.075
GA + AA	290 (67.1)	354 (73.8)	0.716 (0.536-0.956)	0.024^*^

^a^ORs were calculated by unconditional logistic regression and adjusted for age. ^*^ P<0.05.

When stratified by histological type, we found no significant association between genotype and adenocarcinomas risk, but there was a significant relationship between genotypes and squamous cell carcinomas risk (Table 2 and Table 3). The individuals carrying rs1005165 CT and TT had lower risk of squamous cell carcinomas compared to those with CC genotype, and the adjusted ORs were 0.509 (0.310-0.838) and 0.445 (0.228-0.870), respectively. For rs967591, the dominant model (GA+AA genotype) had association with squamous cell carcinomas risk (adjusted OR = 0.611, 95% CI = 0.381-0.978).

**Table 2:** Distribution of genotypes and ORs for adenocarcinomas cases and controls

SNP	Cases (%)	Controls (%)	OR (95% CI)^a^	*P*
rs1005165				
CC	107 (31.1)	119 (25.3)	Ref.	-
CT	170 (49.4)	249 (53.0)	0.785 (0.566-1.089)	0.147
TT	67 (19.5)	102 (21.7)	0.756 (0.504-1.135)	0.177
CT+TT	237 (68.9)	351 (74.7)	0.777 (0.570-1.058)	0.110
rs967591				
GG	108 (31.9)	120 (25.3)	Ref.	-
GA	169 (49.9)	257 (54.2)	0.755 (0.545-1.045)	0.090
AA	62 (18.3)	97 (20.5)	0.736 (0.487-1.113)	0.146
GA + AA	231 (68.1)	354 (73.8)	0.750 (0.550-1.021)	0.068

^a^ORs were calculated by unconditional logistic regression and adjusted for age.

**Table 3:** Distribution of genotypes and ORs for squamous cell carcinomas cases and controls

SNP	Cases (%)	Controls (%)	OR (95% CI)^a^	P^*^
rs1005165				
CC	38 (41.8)	119 (25.3)	Ref.	-
CT	39 (42.9)	249 (53.0)	0.509 (0.310-0.838)	0.008^*^
TT	14 (15.4)	102 (21.7)	0.445 (0.228-0.870)	0.018^*^
CT+TT	53 (58.3)	351 (74.7)	0.491 (0.308-0.782)	0.003^*^
rs967591				
GG	34 (36.6)	120 (25.3)	Ref.	-
GA	43 (46.2)	257 (54.2)	0.613 (0.372-1.011)	0.055
AA	16 (17.2)	97 (20.5)	0.604 (0.314-1.161)	0.131
GA + AA	59(63.4)	354 (73.8)	0.611 (0.381-0.978)	0.040^*^

^a^ORs were calculated by unconditional logistic regression and adjusted for age.^*^ P<0.05. Bold values indicated significance after Bonferroni correction(k = 9).

Table 4 showed the relationship of environmental exposures and NSCLC risk. The subjects with cooking oil fume exposure had higher risk of NSCLC than those without cooking oil fume exposure (OR = 1.571, 95% CI = 1.034-2.385). Gene-environment interactions were also performed. Compared with rs1005165 CT+TT carriers without cooking oil fume exposure, OR of CT+TT carriers with cooking oil fume exposure was 1.797, 95% CI was 1.066-3.031. For rs967591, OR of GA+AA carriers with cooking oil fume exposure was 1.773, 95% CI was 1.055- 2.980, compared with GA+AA carriers without cooking oil fume exposure. The OR of GG carrier with cooking oil fume exposure was higher than the OR of GG carrier without cooking oil fume exposure, but P were larger than 0.05 (Table 5).

**Table 4:** Environmental exposure and NSCLC risk

Environmental factors	Case (%)	Control (%)	χ^2^	*P*	OR (95% CI)
Cooking oil fume exposure	71 (34.6)	56 (25.5)	4.515	0.034	1.571 (1.034-2.385)
Fuel smoke exposure	55 (26.8)	59 (26.6)	0.003	0.953	1.013 (0.660-1.556)
Passive smoking	117 (57.1)	126 (56.8)	0.004	0.974	1.013 (0.690-1.486)

**Table 5:** Interaction of cooking oil fume exposure and rs1005165 and rs967591

		Case (%)	Control (%)	OR (95% CI)^a^	*P*
Cooking oil fume	rs1005165				
-	CT+TT	87 (42.6)	126 (57.5)	Ref.	-
-	CC	46 (22.5)	37 (16.9)	1.710 (1.019-2.868)	0.042
+	CT+TT	46 (22.5)	38 (17.4)	1.797 (1.066-3.031)	0.028
+	CC	25 (12.2)	18 (8.1)	1.755 (0.895-3.440)	0.102
Cooking oil fume	rs967591				
-	GA+AA	90 (44.8)	126 (57.0)	Ref.	-
-	GG	42 (20.9)	39 (17.6)	1.437 (0.855-2.417)	0.172
+	GA+AA	47 (23.4)	38 (17.2)	1.773 (1.055-2.980)	0.031
+	GG	22 (10.9)	18 (8.1)	1.495 (0.752-2.972)	0.252

^a^ORs were calculated by logistic regression and adjusted for age, fuel smoke exposure and passive smoking.

### Polymorphisms and NSCLC overall survival

A total of 283 non-smoking NSCLC female patients were in the cohort study (Table 6). The results of the relationship between these two SNPs and survival time were shown in Table 7. For rs1005165 and rs967591, we found no association between genotypes and survival time. No significant association between genotypes and survival time of patients when stratified by clinical stages or different pathological types (Table 8 and Table 9).

**Table 6:** Demographic and clinical characteristics of subjects in Chinese non-smoking female NSCLC patients

Variables	Cases (%)	MST (mon)	Log-rank *P*	HR (95% CI)
Age				
<60	156 (55.1)	31	<0.001	Ref.
≥60	127 (44.9)	25		1.604 (1.252-2.054)
Clinical stage				
I + II	75 (26.5)	29	0.034	Ref.
III + IV	208 (73.5)	28		1.340 (1.011-1.776)
Operation				
Yes	167 (72.0)	29	0.017	Ref.
No	65 (28.0)	26		1.410 (1.051-1.893)
Adjuvant therapy				
Yes	207 (89.2)	28	0.262	Ref.
No	25 (10.8)	25		1.272 (0.824-1.964)
Pathological type				
Adenocarcinoma	223 (78.8)	28	0.752	Ref.
Squamous cell carcinoma	60 (21.2)	26		1.203 (0.883-1.185)

**Table 7:** Distribution of genotypes and survival time of non-smoking NSCLC female patients

SNP	Cases (%)	MST (mon)	Log-rank *P*	Adjusted HR (95% CI)^a^
rs1005165				
CC	94 (33.5)	28	0.250	Ref.
CT	136 (48.4)	28		1.232 (0.902-1.682)
TT	51 (18.1)	23		1.428 (0.962-2.120)
CT+TT	187 (66.5)	28	0.323	1.283 (0.957-1.719)
rs967591				
GG	89 (32.1)	29	0.258	Ref.
GA	141 (50.9)	28		1.228 (0.896-1.684)
AA	47 (17.0)	23		1.402 (0.927-2.120)
GA+AA	188 (67.9)	27	0.302	1.269 (0.941-1.712)

^a^ HRs were adjusted for age, clinical stage, operation and adjuvant.

**Table 8:** Distribution of genotypes and survival time of patients with different clinical stage

Stage	SNP	Cases (%)	MST (mon)	Log-rank *P*	Adjusted HR (95% CI)^a^
I + II	rs1005165				
	CC	24 (32.0)	26	0.871	Ref.
	CT	37 (49.3)	31		1.234 (0.597-2.555)
	TT	14 (18.7)	25		1.783 (0.761-4.176)
	CT+TT	51 (68.0)	31	0.860	1.363 (0.682-2.723)
	rs967591				
	GG	24 (32.4)	26	0.801	Ref.
	GA	37 (50.0)	31		1.211 (0.586-2.500)
	AA	13 (17.6)	25		1.790 (0.752-4.262)
	GA+AA	50 (67.6)	29	0.890	1.335 (0.668-2.669)
III + IV	rs1005165				
	CC	70 (34.0)	29	0.129	Ref.
	CT	99 (48.1)	28		1.259 (0.880-1.802)
	TT	37 (17.9)	23		1.410 (0.892-2.229)
	CT+TT	136 (66.0)	27	0.147	1.300 (0.929-1.818)
	rs967591				
	GG	65 (32.0)	29	0.188	Ref.
	GA	104 (51.2)	27		1.276 (0.887-1.837)
	AA	34 (16.8)	23		1.367 (0.843-2.218)
	GA+AA	138 (68.0)	27	0.157	1.299 (0.919-1.834)

^a^ HRs were adjusted for age, operation and adjuvant.

**Table 9:** Distribution of genotypes and survival time of patients with different pathological type

Pathological type	SNP	Cases (%)	MST (mon)	Log-rank *P*	Adjusted HR (95% CI)^a^
Adenocarcinoma	rs1005165				
	CC	71 (32.0)	28	0.176	Ref.
	CT	107 (48.2)	29		1.271 (0.877-1.843)
	TT	44 (19.8)	22		1.372 (0.878-2.144)
	CT+TT	151 (68.0)	28	0.490	1.302 (0.920-1.842)
	rs967591				
	GG	70 (32.3)	28	0.203	Ref.
	GA	108 (49.8)	28		1.294 (0.890-1.883)
	AA	39 (17.5)	22		1.332 (0.829-2.140)
	GA+AA	147 (67.7)	28	0.522	1.304 (0.915-1.860)
Squamous cell carcinoma	rs1005165				
	CC	23 (39.0)	30	0.589	Ref.
	CT	29 (49.2)	26		1.237 (0.643-2.380)
	TT	7 (11.9)	25		0.942 (0.341-2.605)
	CT+TT	36 (61.1)	26	0.448	1.169 (0.627-2.179)
	rs967591				
	GG	19 (31.7)	31	0.467	Ref.
	GA	33 (55.0)	26		1.391 (0.704-2.746)
	AA	8 (13.3)	25		1.118 (0.420-2.979)
	GA+AA	41 (68.3)	26	0.297	1.322 (0.694-2.520)

^a^ HRs were adjusted for age, clinical stage, operation and adjuvant.

## DISCUSSION

In this study, we investigated the association between genetic polymorphisms of PPP1R13L rs1005165 and CD3EAP rs967591 with NSCLC risk and survival in Chinese non-smoking females.

The results suggested that rs1005165 C>T and rs967591 G>A were associated with lower NSCLC risk. The homozygous wild genotype carriers had higher NSCLC risk than heterozygote carriers and homozygous mutant genotype carriers. Xiao M et al [18] found that both minor T allele in PPP1R13L rs1005165 and minor A allele in CD3EAP rs967591 were associated with the lower levels of BPDE-adducts which was associated with the alteration of repair efficiency to DNA damage caused by Benzo[a]pyrene. This suggested that T allele in rs1005165 and A allele in rs967591 might contribute to enhance predictive value for individual’s DNA repair capacity in response to environmental carcinogens, such as cooking oil fume, fuel smoke and passive smoking exposure. This suggested that the two SNPs and environmental factors might have interactive function in NSCLC occurrence. In our study, OR of CT+TT carriers with cooking oil fume exposure was 1.797, 95% CI was 1.066-3.031, compared with rs1005165 CT+TT carriers without cooking oil fume exposure; OR of rs967591 GA+AA carriers with cooking oil fume exposure was 1.773, 95% CI was 1.055-2.980, compared with GA+AA carriers without cooking oil fume exposure. The OR of GG carrier with cooking oil fume exposure was higher than the OR of GG carrier without cooking oil fume exposure, but P was larger than 0.05. The reason of not completely conformity might be small sample size in our study. Yin JY et al [19] found that A-allele for CD3EAP rs967591 was associated with increased lung cancer risk of 79 nonsmoking Chinese female lung cancer cases and 108 cancer-free controls, which had the opposite conclusion. The reason might be different histologic types of cases in that study. There were also some investigations about the relationship of the two SNPs and cancer risk [20-24], but the results were not same. In view of such different results, the relationship between the two SNPs and NSCLC should be confirmed by larger sample size population study and functional analyses.

We also investigated the relationship between PPP1R13Lrs1005165 or CD3EAP rs96759 and non-smoking NSCLC females’ survival, and found no association. However, other studies showed that rs1005165 and rs967591 were associated with survival of early-stage lung cancer [17, 25]. Rs1005165 T allele and rs967591 A allele exhibited a worse overall survival. The rs967591A allele had significantly higher activity of the CD3EAP promoter compared with the rs967591G allele, but rs967591 did not have effect on the activity of PPP1R13L promoter, and rs967591 was associated with the level of CD3EAP mRNA expression in lung tissues [26]. These different conclusions were likely to be caused by difference of clinical stage. In our study, 73.5% patients were III and IV NSCLC patients. The different conclusions might also be caused by different proportion of histologic type. PPP1R13L was predicted to be associated with cancer progression and response to therapy, and sqamous cell carcinoma was more sensitive to adjuvant therapy than adenocarcinoma. 62.1% patients in that study were sqamous cell carcinoma, but only 21.2% cases were sqamous cell carcinoma in our study. The effects of the polymorphisms rs1005165 on gene function are not known. The two SNPs have also been studied in relation to prognosis and therapy for different cancers [16, 27-29], but the results were not consistent; the reason might be different cancers and different ethnics.

In conclusion, findings from this study suggested that PPP1R13L rs1005165 and CD3EAP rs967591 might be associated with NSCLC risk in Chinese non-smoking females, but no significant correlation between the two SNPs and NSCLC survival time was found.

## MATERIALS AND METHODS

### Study subjects

The first part of this study was a hospital-based case-control study, which consisted of 442 NSCLC cases and 480 cancer-free controls (collected from April 2011 to April 2014). All participants were non-smoking females and unrelated ethnic Han Chinese. Individuals smoking less than 100 cigarettes in lifetime were defined as non-smokers. The patients were from Liaoning Cancer Hospital and Liaoning Northern Hospital. All of the patients were newly diagnoses with histopathologically confirmed NSCLC and without previous treated. Controls were selected from medical examination centers in the same hospitals during the same period, who were matched to cases on age (±5 years). A face-to-face questionnaire interview was performed to collect demographics and environmental exposure information, including age, cooking oil fume exposure, fuel smoke exposure and passive smoking. Among the cases in above case-control study, 303 non-smoking primary NSCLC female patients collected from April 2011 to April 2013were in the cohort study. The follow-up time was terminated in April 2015. The survival information of the patients were collected from telephone follow-up every 3 months, death was defined as the outcome event. Overall survival was calculated from the date of diagnosis to the date of death or the end of patient follow-up. 20 patients were lost during the follow-up period, and the lost rate was 6.6%. This study was approved by the Institutional Review Board of China Medical University.

### Genotyping

Genomic DNA was extracted from 1 ml samples of whole blood by phenol-chloroform method. Genotyping was performed on an Applied Biosystems 7500 FAST Real-time PCR System using a TaqMan SNP genotyping assay (Applied Biosystems, Foster City, CA). 10% of the samples were randomly selected to be re-tested for quality control.

### Statistical analysis

Statistical analyses were performed by SPSS 22.0 (IBM SPSS Statistics, IBM). All of the tests were two-sides and statistical significance was defined as P<0.05. Hardy-Weinberg equilibrium was tested by Pearson chi-squared test. Differences between cases and controls were calculated by t-test for continuous variables or chi-squared test for categorical variables. Odds ratios (ORs) and their 95% confidence intervals (CIs) were calculated by logistic regression (adjusted for age) to determine the correlation between SNPs and NSCLC risk. Kaplan-Meier method and log-rank test were performed to assess the relationship between different genotypes and OS. Hazard ratios (HRs) and their 95% CIs for OS were calculated by multivariate Cox proportionally hazards model, and co-variants were age, clinical stage and treatment information.
